# Supplementary material for: Personalised Tasted Masked Chewable 3D Printed Fruit-Chews for Paediatric Patients
Source: Pharmaceutics. 2021 Aug 20;13(8):1301. doi: 10.3390/pharmaceutics13081301 (PMC8400795; doi:10.3390/pharmaceutics13081301)
Supplement: Supplementary file 1 [file pharmaceutics-13-01301-s001.zip › pharmaceutics-1320257-supplementary.pdf]

pharmaceutics

Supplementary Materials: Personalised Tasted Masked Chewable 3D Printed Fruit-Chews for Paediatric Patients

Atabak Ghanizadeh Tabriz, Daniel Henri George Fullbrook, Lilian Vilain, Youri Derrar, Uttom Nandi, Clara Grau, Anaïs Morales, Gemma Hooper, Zoltan Hiezl and Dennis Douroumis

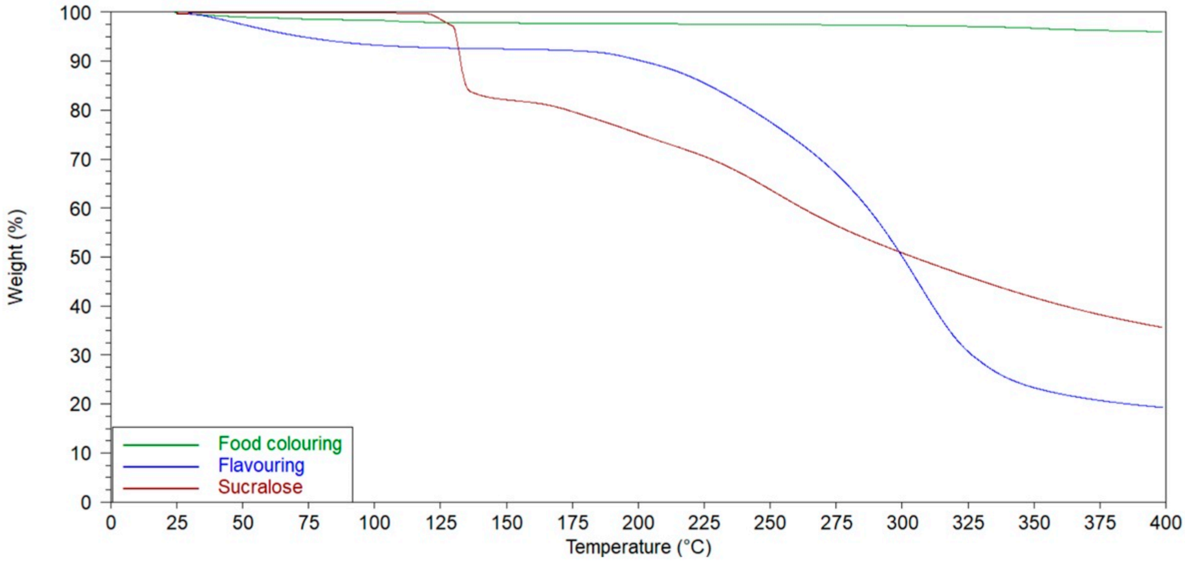

Figure S1. TGA thermograms of food colouring, straweverry falvour and sucralose.

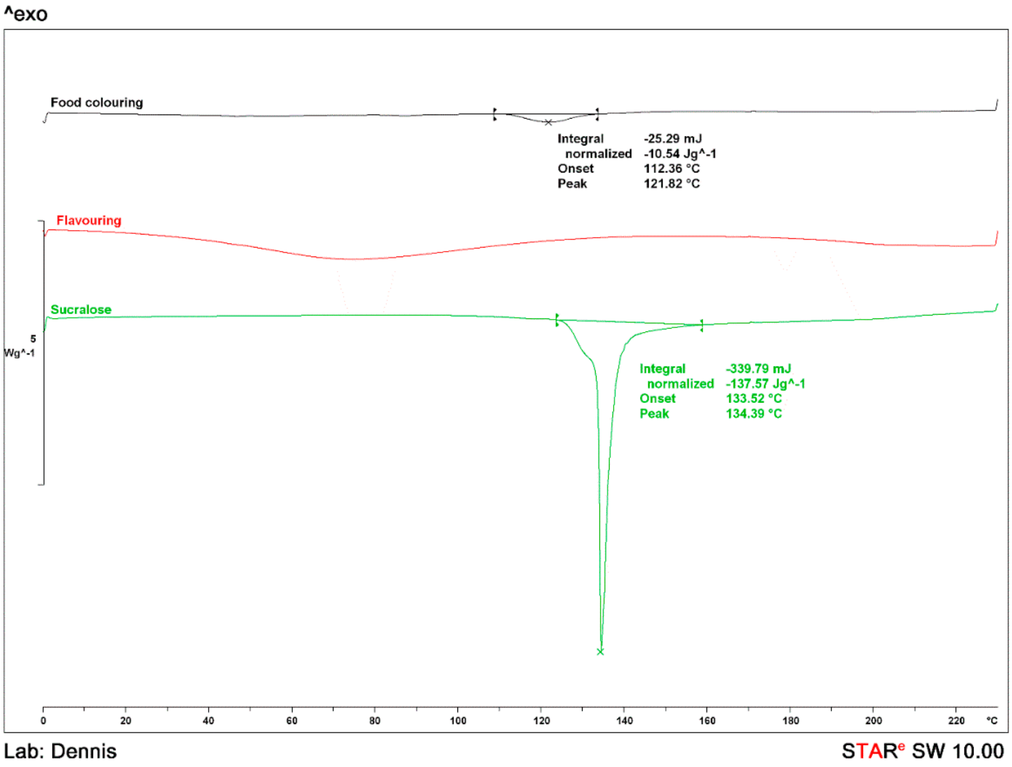

Figure S2. DSC thermogras of food colouring, straweverry falvour and sucralose.

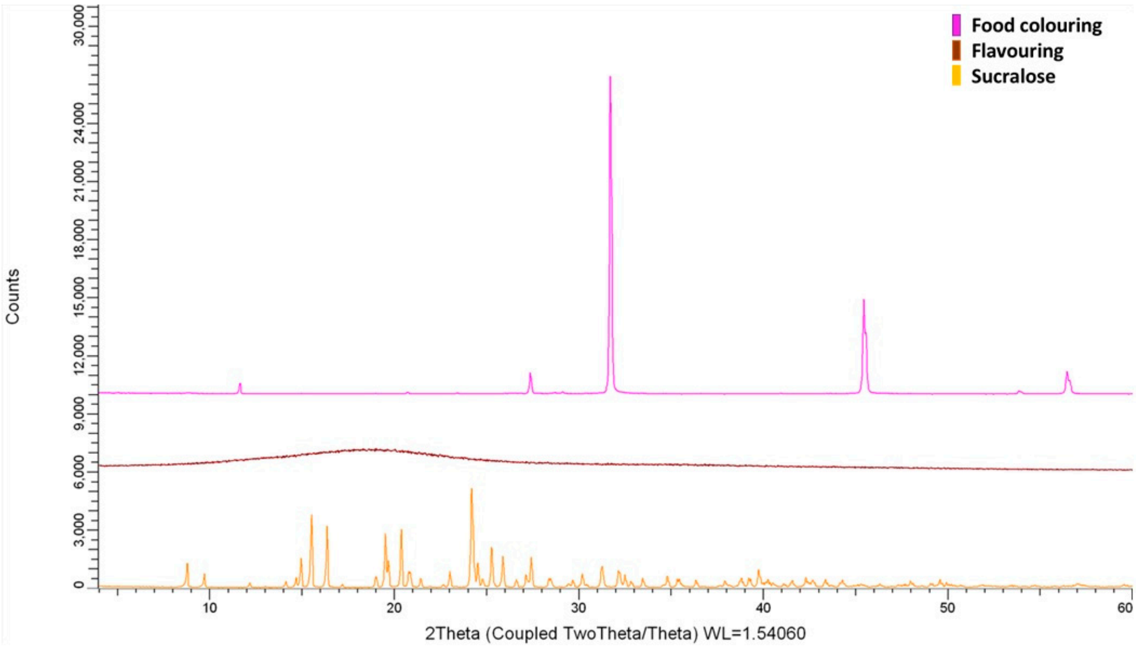

Figure S3. XRD patterns of food colouring, straweverry falvour and sucralose.
